# Patients with type 2 diabetes present with multiple anomalies of the pancreatic arterial tree on abdominal computed tomography: comparison between patients with type 2 diabetes and a matched control group

**DOI:** 10.1186/s12933-020-01098-1

**Published:** 2020-08-05

**Authors:** Laure Alexandre-Heymann, Matthias Barral, Anthony Dohan, Etienne Larger

**Affiliations:** 1 Service de Diabétologie, Hôpital Cochin, 123 Boulevard de Port Royal, 75014 Paris, France; 2 Département Hospitalo Universitaire, INSERM U1016, Université Paris Descartes, Paris, France; 3 Service de Radiologie A, Hôpital Cochin, Paris, France; 4 Université de Paris, Paris, France

**Keywords:** Atherosclerosis, Pancreas density, Pancreas ischemia, Pancreas perfusion, Pancreas volume, Type 2 diabetes

## Abstract

**Background:**

Studies suggest that cardio-vascular risk factors could foster the development of type 2 diabetes (T2D). This could partly be mediated by pancreatic atherosclerosis resulting in pancreatic ischemia. We hypothesized that patients with T2D present with more severe atherosclerosis of pancreas-bound arteries than control patients without T2D.

**Methods:**

We performed a retrospective study comparing the abdominal computed tomography of patients with T2D and of control subjects matched for gender and for age. We performed a multivariate logistic regression with adjustment for age, gender, BMI and the presence or absence of hypertension.

**Results:**

Forty-eight patients with T2D and 48 control subjects were included. A calcification score of the splenic artery was defined (from 0: no calcification to 3: continuous linear calcifications). Seventeen percent of the patients with T2D presented with a high calcification score (i.e. 2 or 3), versus only 2% of the control subjects (p = 0.04). The mean number of pancreas-bound branches among the greater pancreatic artery, dorsal pancreatic artery and inferior pancreatic artery (from 0 to 3) was lower in patients with T2D than in control subjects (1.1 vs 1.7, p = 0.003). The mean number of visible intrapancreatic arterial subdivisions (from 0 to 2) was lower in patients with T2D than in control subjects (0.7 vs 1.3, p = 0.0017). All these differences hold true using multivariate logistic regression. None of these differences correlated with the duration of diabetes. The relationship between pancreas volume and BMI seen in control subjects was not confirmed in patients with T2D. Conversely, in patients with T2D but not in control subjects, the splenic artery diameter correlated with the pancreas volume.

**Conclusions:**

Patients with T2D present with more calcifications of the splenic artery and with a less dense pancreatic arterial tree than control subjects.

## Background

Vascular lesions are very common in patients with type 2 diabetes, resulting in various complications [1]. The causal link between type 2 diabetes and micro- and macro-vascular lesions is widely accepted. Conversely, several epidemiological and experimental studies suggest that cardio-vascular anomalies could foster the development of type 2 diabetes: patients who suffer from hypertension, myocardial infarction, or arterial stiffness as measured by the carotid-femoral pulse wave velocity [2–4], and patients who smoke [5, 6] are all more at risk of type 2 diabetes than control subjects or than non-smoker patients. Patients with an increased carotid intima-media thickness, a marker of cardio-vascular risk, are more frequently glucose intolerant [7, 8]. Moreover, in the NAVIGATOR study that included patients with impaired glucose tolerance and cardiovascular disease or risk factors, treatment with an angiotensin II receptor antagonist for 5 years decreased the incidence of diabetes [9].

These correlations could partly be mediated by pancreatic atherosclerosis through pancreatic ischemia. Indeed, in vitro, insulin secretion decreases with hypoxia [10] and beta cells show signs of dedifferentiation in hypoxic conditions [11]. Moreover, it has been known for more than a century that atherosclerosis and other vascular anomalies are more often seen in the intrapancreatic vessels of patients with diabetes than in control subjects [12, 13]. A recent study showed that the extent of calcifications in the superior mesenteric artery associates with the presence of diabetes [14]. In a postmortem angiography study, it was shown that vascular anomalies are more often seen in patients with type 2 diabetes than in control subjects, independently from the duration of diabetes [15].

As a reminder, the body and tail of the pancreas are mainly irrigated by a branch of the celiac trunk, the splenic artery, that usually divides into the dorsal pancreatic artery, the inferior pancreatic artery, the greater pancreatic artery and other pancreatic branches. The head of the pancreas is mainly irrigated by branches arising from the gastroduodenal and the superior mesenteric arteries. All these branches are highly anastomosed [16–18].

We hypothesized that patients with type 2 diabetes more often present with vascular abnormalities of the pancreas-bound arteries than control subjects. Hence, the aim of this study was to compare the pancreatic vascular density of patients with type 2 diabetes and of control subjects.

## Methods

### Aim

The primary aim of our study was to quantify and compare the vascular anomalies of pancreas-bound arteries of patients with type 2 diabetes and of control subjects. The secondary objective was to compare the volume and density of the pancreas between the two groups.

### Design and setting

We performed a retrospective monocentric study in a tertiary referral center for type 2 diabetes (Cochin Hospital, Paris), comparing patients with type 2 diabetes and matched control subjects. This was a retrospective study, using existing clinical data only.

Patients were included if they were older than 18 years old, had type 2 diabetes, and had had an abdominal CT before and after iodinated contrast media intravenous injection between January and December 2017 in our institution. Using the data available from a study performed on postmortem pancreatic angiography [15], we estimated that we needed at least 39 subjects in each group in order to detect a difference between the groups (OR of decreased vascularity in patients with type 2 diabetes versus in control subjects: 5.4, expected proportion of exposed controls: 20%, alpha risk: 0.05, study power: 90%).

The list of patients with type 2 diabetes was extracted from our electronic medical records system by selecting patients who had an International Classification of Disease code for type 2 diabetes (ICD code: e11xx) and a billing code of abdominal CT scan. Non-inclusion criteria were as follows: CT scans performed without contrast media injection, or CT scans acquired only after injection of contrast media (hence lacking images with or without contrast, respectively). 98 patients out of 223 were pre-selected at random in order to retain enough patients after exclusion.

Exclusion criteria were as follows: type 1 diabetes or other causes of diabetes mellitus (MODY, cystic fibrosis…); known pancreatic disease (acute pancreatitis in the 6 months prior to the CT scan, chronic pancreatitis, pancreatic adenocarcinoma, history of pancreatic surgery); liver or biliary disease (hepatocellular carcinoma, advanced cirrhosis, cholangiocarcinoma, biliary prosthesis); cardiorespiratory arrest or multi-visceral failure in the month prior or in the week following the CT scan; lack of recent medical reports (maximum 2 years). After exclusion, 48 patients with type 2 diabetes were finally included in the study.

Control subjects were selected from the list of patients for whom an abdominal CT before and after iodinated contrast media intravenous injection had been performed from January 1st 2016 to December 31st 2017 in our hospital. The total number of potentially eligible control patients was 3267. For each included patient with type 2 diabetes, a control subject was selected. The control subjects were matched with the patients for gender and for age ± 1 year.

For each patient with T2D, a first potentially eligible control subject was picked at random. The medical records of the eligible control subjects were then assessed in order to check for the presence or absence of exclusion criteria. If the first eligible control subject was excluded, the records of another potentially eligible control were assessed, etc. 177 potentially eligible control subjects were excluded (Additional file 1: Table S1).

The exclusion criteria were the same for the patients and for the control subjects. A supplementary exclusion criterion for control subjects was diabetes of any kind. The medical files of all control subjects were checked to ensure that they did not have diabetes, i.e. that they did not have a history of fasting hyperglycemia (> 7 mmol/L) or of hyperglycemia at any time (> 11 mmol/L) and that they did not take any antidiabetic medication.

### Processes, interventions and comparisons

#### Clinical and biological characteristics

All clinical and biological data were extracted from the subjects’ electronic medical records.

The presence or absence of hypertension, dyslipidemia, and coronary disease was recorded for all the subjects. Hypertension was deemed present if a history of hypertension was noted in the patient’s medical record AND/OR if the patient was taking any antihypertensive medication.

Dyslipidemia was deemed present if a history of dyslipidemia, hypercholesterolemia or hypertriglyceridemia was noted in the patient’s medical record AND/OR if the patient was taking statin or fibrate medication AND/OR if the patient had a known history of LDL cholesterol concentration > 4.95 mmol/L or a known history of serum triglyceride concentration > 1.71 mmol/L.

Coronary disease was deemed present if a history of coronary disease, myocardial infarction, coronary angioplasty or coronary bypass surgery was noted in the patient’s medical record.

Serum creatinine concentration was recorded. Estimated glomerular filtration rate (eGFR) was calculated using MDRD formula [19]. Renal failure was defined as an eGFR < 60/mL/min/1.73 m^2^. Severe renal failure was defined as an eGFR < 30 mL/min/1.73 m^2^.

For the patients with type 2 diabetes, the presence or absence of peripheral neuropathy, nephropathy and retinopathy was recorded. Peripheral neuropathy was deemed present if a history of peripheral neuropathy was noted as such in the patient’s medical record, AND/OR if lower limbs paresthesia, lower limb neuropathic pain, or abnormalities at the monofilament testing were noted in the patient’s medical record.

Nephropathy was deemed present if a history of diabetic nephropathy was noted as such in the patient’s medical record AND/OR if there was a history of microalbuminuria > 3 mg/mmol of creatininuria or a history of proteinuria, or if the MDRD glomerular filtration rate was < 60 mL/min without another known cause for renal dysfunction.

Retinopathy was deemed present if a history of diabetic retinopathy was noted as such in the patient’s medical record AND/OR if there was a history of macular edema or of retinal photocoagulation.

#### Computed tomography

All the patients had abdominopelvic computed tomography (CT) using a Somatom Sensation 64^®^ (Siemens Healthcare, Forchheim Germany). The following scanner parameters were used: 279–450 mm field of view, 38.4 mm beam collimation (64 × 0.6 mm collimator setting), 120 peak kVp tube potential, 0.5–0.8 s gantry revolution time and 46 mm per gantry rotation table speed resulting in a beam pitch of 1.2 and 310–500 mm field of view, and 55 mm per gantry rotation table speed resulting in a beam pitch of 1.38. Online, real time, anatomy-adapted, attenuation-based tube current modulation techniques (Care Dose 4D^®^, Siemens Medical Solution) were used with a tube current set to 120–170 effective mAs. At the start of the procedure, 120 mL of non-ionic iodinated contrast material (Iomeprol, Iomeron^®^, Bracco Imaging SpA, Milan, Italy or iopamidol, Iopamiron^®^, Guerbet, Roissy-Charles de Gaulle, France) were injected intravenously through a 20-Gauge catheter into an antecubital vein, at the rate of 3 mL/s by using an automated power injector. Two pass imaging sets were obtained 25 s and 70 s after the start of the contrast material administration. All CT examinations were performed from the hepatic dome to the lower margin of the symphysis pubis, using a cephalocaudal direction after breath hold instruction was given.

After acquisition, CT data were reconstructed at 1 mm thickness at 0.5 mm intervals for transverse and multiplanar reconstructions and 3D imaging. All data were stored on internal picture archiving and communication system (PACS, Directview, V12.1.5.1156, Carestream Health Inc., Rochester, NY, USA).

#### Pancreas volume

**Fig. 1:**
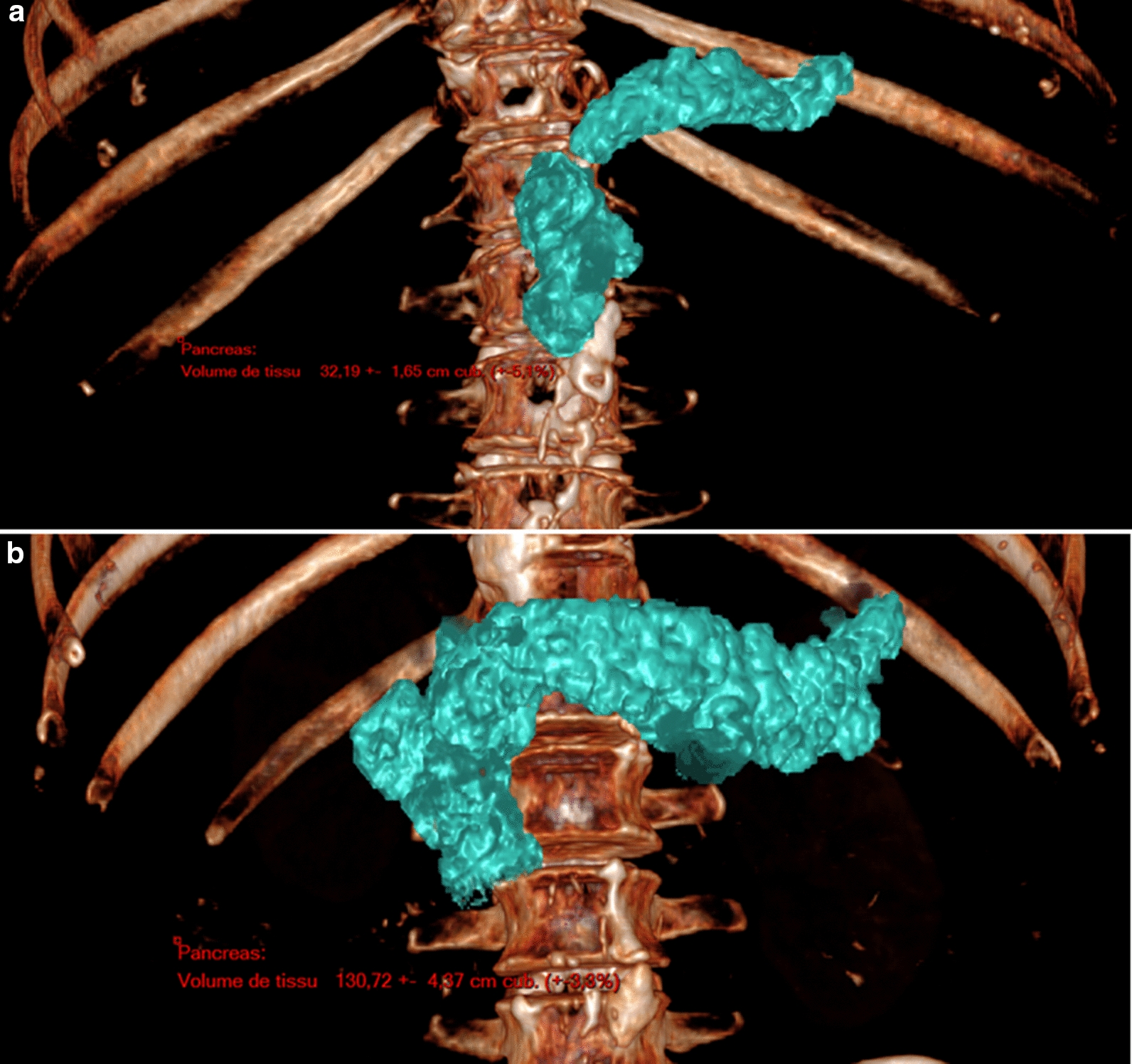
Three-dimensional computed tomography image of the pancreas. **a** Calculation of the pancreatic volume reveals a volume of 32 ± 1.7 cm^3^. **b** Calculation of the pancreatic volume reveals a volume of 131 ± 4 cm^3^

For all patients, transverse CT images were analyzed along with multiplanar and 3D images using the PACS workstation. Pancreas volume calculation (Fig. 1) was performed on venous phase CT images using a combination of contour drawing, thresholding and region growing. Interpolation between the marked slices was performed semi-automatically using a linear algorithm [20, 21]. The splenic, mesenteric and hepatic arteries, celiac trunk, splenic vein, and the superior mesenteric vein were excluded from pancreatic segmentation.

#### Vessels assessment

**Fig. 2:**
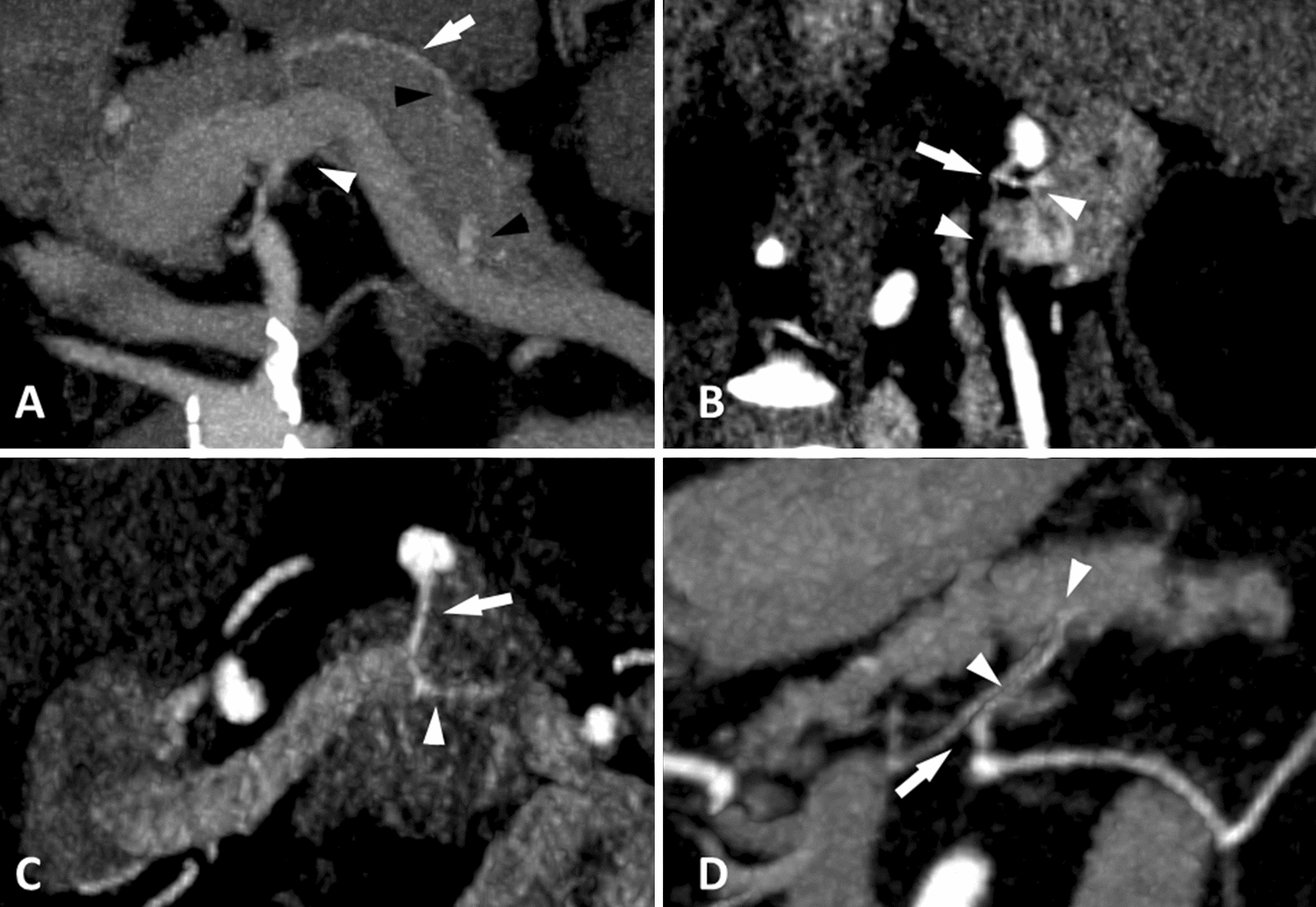
Multi-detector computed tomography after intravenous administration of iodinated contrast material at the arterial phase. **a** Maximum intensity projection in the transverse plane demonstrates pancreatic transverse (*arrow*) artery communicating with the greater pancreatic artery (*arrowhead*) with first- and second-order vessel division (*black arrowheads*). **b** Multiplanar oblique reconstruction reveals a dorsal pancreatic artery (*arrow*) with first- and second-order vessel division (*arrowheads*). **c** Maximum intensity projection with oblique reconstruction demonstrates greater pancreatic artery (*arrow*) with first order vessel division (*arrowhead*). **d** Maximum intensity projection with oblique reconstruction reveals inferior pancreatic artery (*arrow*) with first- and second order vessel division (*arrowheads*)

The vessel assessment (Fig. 2) was performed on arterial phase CT images. The diameter of the splenic, hepatic and gastroduodenal arteries was measured one centimeter after their origin. The presence and the total number of visible pancreas-bound branches emerging from the splenic artery or sometimes from the celiac trunk or from the superior mesenteric artery in case of anatomical variations (i.e. the greater pancreatic artery, the dorsal pancreatic artery and the inferior pancreatic artery) were noted (from 0 to 3 visible branches). Their diameters were measured one centimeter after their origin. Simultaneously, the identification of intrapancreatic first- and second-order vessel division was performed.

#### Calcifications

**Fig. 3:**
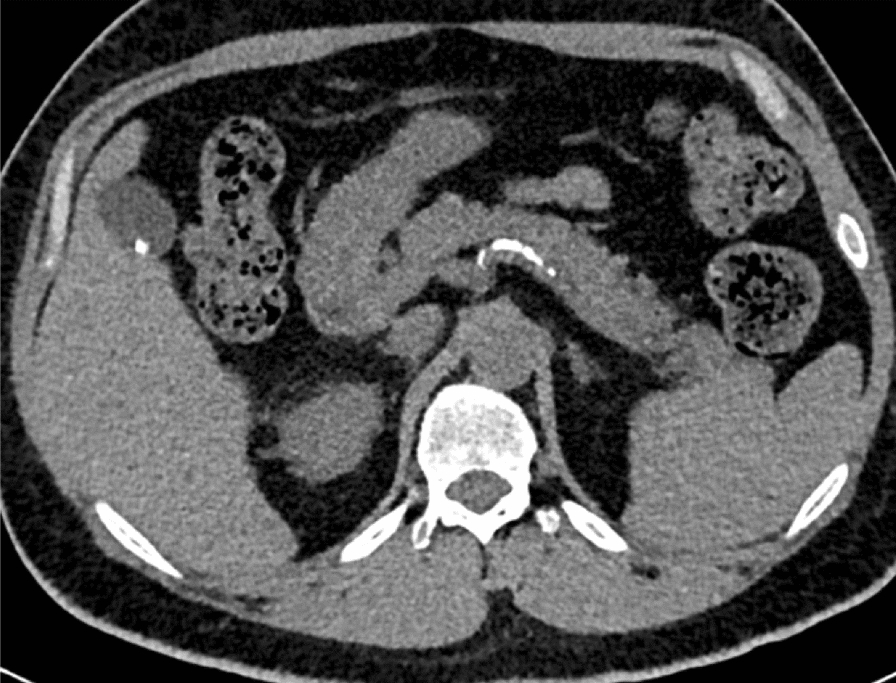
Multi-detector computed tomography without contrast. Calcifications in the splenic artery’s wall in the pancreas parenchyma

Calcifications in the splenic artery and abdominal aorta walls were evaluated on the images without contrast and classified according to the following score:

0: no calcification; 1: scarce interspersed calcifications; 2: linear calcifications with intervals of normal artery; 3: circumferential calcifications for the abdominal aorta, and continuous linear calcifications for the splenic artery (Fig. 3).

#### Pancreas and liver density

Pancreas and liver density were quantified on the images without contrast. The density of the pancreas, in Hounsfield Units (HU), was defined as the mean density of 3 Regions Of Interest (ROI) delineated in the head, body and tail of the pancreas, respectively. Each ROI was carefully delineated in order to exclude vessels and calcifications.

#### Interobserver reliability

All CT images analyses were performed by a unique investigator blinded to the diabetes or control status of the subjects. To validate our image analysis methods, two different sets of randomly selected patients were quantified by a second, independent investigator, who was also blinded to the diabetes or control status of the subjects.

The characteristics of the pancreas (pancreas density and volume) were validated in a subgroup of 21 random subjects (11 patients with type 2 diabetes and 10 control subjects).

The characteristics of the vessels (abdominal aorta and splenic artery calcium scores, splenic artery diameter, number of pancreas-bound arteries and of intrapancreatic arterial subdivisions) were validated in another subgroup of 22 random subjects (11 patients with type 2 diabetes and 11 control subjects).

#### Duration of diabetes

In order to know if the characteristics of the pancreas observed in patients with type 2 diabetes correlated with the duration of diabetes, we divided the patients in whom the duration of diabetes was known into two subgroups: the patients of the first group had a duration of diabetes of 0 to 10 years, and the patients of the second group had a duration of diabetes of more than 10 years. Age at first diagnosis of diabetes, from which diabetes duration was determined, was established according to patients and/or physician reports, noted in the medical records of the patients. We then compared the characteristics of the pancreas and its vessels between the two subgroups.

### Statistical analysis

Data are presented as mean with standard deviation (SD) for the normally distributed variables, and as median with interquartile range (IQR) for the variables with non-normal distribution. For continuous variables, t-tests (paired or unpaired) were used when 2 groups of normally distributed variables were compared, Mann-Whitney test was used when 2 groups of unpaired variables with non-normal distribution were compared, Wilcoxon matched-pair signed rank test was used when 2 groups of paired variables with non-normal distribution were compared. ANOVA was used when more than 2 groups of normally distributed variables were compared, Kruskal–Wallis comparison was used when more than 2 groups of variables with non-normal distribution were compared.

For categorical variables, McNemar test was used when 2 paired groups were compared, Fisher’s exact test was used when 2 unpaired groups were compared, and Chi Square was used when more than 2 groups were compared.

After performing a log likelihood ratio comparison of different models, we tested if there was an independent association between the presence or absence of type 2 diabetes and pancreas and liver characteristics using multivariate logistic regression with adjustment for age, gender, BMI and the presence or absence of hypertension. We did the same to test the association between a duration of diabetes of less or more than 10 years and pancreas and liver characteristics.

Interobserver reliability was assessed using intraclass correlation coefficient for continuous variables, and Cohen’s weighted kappa for categorical variables.

Analyses were performed using Graphpad Prism 5 (GraphPad Software, Inc., San Diego, CA) and the Real Statistics Resource Pack software (Release 6.8), copyright (2013–2020) (www.real-statistics.com).

## Results

### Characteristics of patients

Forty-eight patients with type 2 diabetes and 48 control subjects were included and analyzed. Mean age was 64.7 years-old and 65% of the subjects were male. Mean Body Mass Index (BMI, defined as the weight in kilograms divided by the height in meters squared) was higher in the patients with type 2 diabetes than in the control subjects (27.7 kg/m^2^ vs 23.8 kg/m^2^, p < 0.001). The patients with type 2 diabetes presented more often with hypertension, hypercholesterolemia and history of coronary disease than the control subjects. In patients with type 2 diabetes, the median HbA_1c_ was 7.5% (58 mmol/mol), and the median duration of diabetes was 10 years (Table 1).

**Table 1:** Clinical characteristics of the subjects

	Patients with T2D N = 48	Control subjects N = 48	p
General characteristics			
Male, *n* (*%*)	31 (65)	31 (65)	1
Age, years: *mean* (*SD*)	64.8 (12.8)	64.7 (12.8)	0.2
BMI, kg/m^2^*: mean* (*SD*)^a^	27.7 (5.9)	23.8 (4.9)	< 0.001
Cardiovascular risk factors			
Hypertension, *n* (*%*)^b^	30 (63)	13 (29)	0.013
Dyslipidemia, *n* (*%*)^c^	32 (76)	11 (31)	< 0.0001
Coronary disease, *n* (*%*)^d^	13 (28)	4 (9)	0.05
Estimated glomerular filtration rate, mL/min: *mean* (*SD*)^*e*^	85.9 (30.3)	94.9 (23.6)	0.2
Renal failure, *n* (*%*)^e^	9 (19)	3 (7)	0.2
Severe renal failure, *n* (*%*)^e^	1 (2)	0 (0)	1
Tobacco use (current or stopped for less than 3 years), *n* (*%*)^f^	11 (25)	6 (19)	0.6
Diabetes characteristics			
HbA_1c_, mmol/mol: *median* (*IQR*)^g^	58 (48–76)	34 (33–42)	NA
HbA_1c_, *%: median* (*IQR*)^g^	7.5 (6.5–9.1)	5.3 (5.2–6)	NA
Diabetes duration, years: *median* (*IQR*)^h^	10 (5.5–17.3)		
Insulin use, *n* (*%*)	19 (40)		
Neuropathy, *n* (*%*)^i^	15 (52)		
Nephropathy, *n* (*%*)^j^	15 (47)		
Retinopathy, *n* (*%*)^k^	11 (42)		

*T2D* type 2 diabetes, *BMI* body mass index, *SD* standard deviation, *IQR* interquartile range

^a^Available in 45 patients with T2D and 35 control subjects

^b^Available in 47 patients with T2D and 45 control subjects

^c^Available in 42 patients with T2D and 35 control subjects

^d^Available in 46 patients with T2D and 44 control subjects

^e^Glomerular filtration rate as evaluated by MDRD. Renal failure defined as a GFR < 60 mL/min/1.73 m^2^. Severe renal failure defined as a GFR < 30 mL/min/1.73 m^2^. Available in 48 patients with T2D and 46 control subjects

^f^Available in 44 patients with T2D and 32 control subjects

^g^Last HbA_1c_ in the medical file. Available in 34 patients with T2D and 5 control subjects

^h^Available in 32 patients

^i^Available in 29 patients

^j^Available in 32 patients

^k^Available in 26 patients

The abdominal CT were mainly performed for the following indications: gastro-intestinal or urinary symptoms, e.g. abdominal pain, diarrhea, bowel obstruction or urinary urgencies (35% of the indications in patients with type 2 diabetes versus 46% in control subjects); diabetes characterization (23% of the indications in patients with type 2 diabetes); cancer extension work-up or cancer follow-up (6% of the indications in patients with type 2 diabetes versus 29% in control subjects); and asthenia or loss of body weight (13% of the indications in patients with type 2 diabetes versus 2% in control subjects) (Table 2).

**Table 2:** Abdominal computed tomography indications

	Patients with T2D N = 48	Control subjects N = 48	p
Gastro-intestinal or urinary symptoms *n* (*%*)	17 (35)	22 (46)	0.4
Diabetes characterization n (*%*)	11 (23)	NA	NA
Cancer extension work-up or cancer follow-up *n* (*%*)	3 (6)	14 (29)	0.02
Asthenia or loss of body weight *n* (*%*)	6 (13)	1 (2)	0.1
Back pain/aortic dissection suspicion *n* (*%*)	3 (6)	1 (2)	0.6
Abdominal mass characterization (non cancerous) *n* (*%*)	1 (2)	5 (10)	0.2
Unexplained fever *n* (*%*)	1 (2)	2 (4)	1
Abdominal surgery complication *n* (*%*)	2 (4)	2 (4)	0.6
Car accident *n* (*%*)	1 (2)	1 (2)	0.5
Anemia work-up *n* (*%*)	1 (2)	0	1
Resistant hypertension work-up *n* (*%*)	2 (4)	0	0.5

*T2D* Type 2 diabetes

### Interobserver reliability of image analysis

Interobserver reliability was assessed. Cohen’s weighted kappa was as follows: for abdominal aorta calcium score: 0.69 (0.48–0.90), for splenic artery calcium score: 0.81 (0.55–1), for number of pancreas-bound arteries: 0.56 (0.34–0.79) and for number of intrapancreatic arterial subdivisions: 0.61 (0.35–0.88).

Intraclass correlation coefficient was as follows: for pancreas volume: 0.74 (0.44–0.89), for pancreas density: 0.87 (0.68–0.95) and for splenic artery diameter: 0.60 (0.27–0.79).

### Vessels assessment

#### Calcification scores

As stated above, the calcifications in the splenic artery and abdominal aorta walls were assessed on the images without contrast. They were scored from 0 (uncalcified artery) to 3 (continuous linear calcifications in the splenic artery or circumferential calcifications in the abdominal aorta).

We assessed the calcifications in the splenic artery as it is the major provider of arterial blood to the body and tail of the pancreas. The patients with type 2 diabetes more often presented with a high calcification score of the splenic artery than the control subjects. Conversely, the splenic artery walls were less often free of calcifications in the patients with type 2 diabetes than in the control subjects (Table 3).

**Table 3:** Pancreas and liver assessment

	Patients with T2D N = 48	Control subjects N = 48	p (univariate)	p (multivariate)
Splenic artery calcium score *n* (*%*)				
0	31 (65)	39 (81)	0.04	0.035
1	9 (19)	8 (17)		
2 or 3	8 (17)	1 (2)		
Abdominal aorta calcium score, *n* (*%*)				
0	4 (8)	15 (31)	0.019	0.009
1	23 (48)	17 (35)		
2 or 3	21 (44)	16 (33)		
Number of pancreas-bound branches, *n* (*%*)				
0	19 (40)	6 (12)	0.003	0.036
1	9 (19)	9 (19)		
2 or 3	20 (41)	33 (69)		
Number of intrapancreatic arterial subdivisions, *n* (*%*)				
0	26 (54)	9 (19)	0.001	0.008
1	10 (21)	14 (29)		
2	12 (25)	25 (52)		
Pancreas volume, cm^3^: *mean* (*SD*)	61.5 (32.8)	60.7 (26.0)	0.9	0.3
Pancreas density, *Hounsfield units: median* (*IQR*)^a^	27.5 (20.5–32.6)	34.3 (25.6–39.1)	0.026	0.5
Liver density, *Hounsfield units: median* (*IQR*)	46.5 (38.3–51.8)	53.0 (46.3–56)	0.0001	0.1

Multivariate logistic regression adjusted for age, gender, BMI and presence or absence of hypertension

*T2D* Type 2 diabetes, *SD* standard deviation, *IQR* interquartile range

^a^Pancreas density defined as the mean of 3 ROIs from the head, body and tail of the pancreas

As for the abdominal aorta, the patients with type 2 diabetes more often presented with a high calcification score than the control subjects. Conversely, the abdominal aorta walls were less often free of calcifications in the patients with type 2 diabetes than in the control subjects (Table 3).

#### Pancreas-bound branches and their intrapancreatic divisions

The presence of the greater pancreatic artery, the dorsal pancreatic artery and the inferior pancreatic artery on CT images was assessed, and their total number (from 0 to 3) was noted. The mean number of these branches was lower in patients with T2D than in control subjects (1.1 vs 1.7, p = 0.003). No pancreas-bound branch was seen in 40% of the patients with type 2 diabetes versus in 13% of the control subjects (Table 3).

Moreover, the intrapancreatic first- and second-order vessel divisions were assessed. The mean number of visible intrapancreatic arterial subdivisions (from 0 to 2) was lower in patients with T2D than in control subjects (0.7 vs 1.3, p = 0.0017). Subdivisions were not seen at all in 54% of patients with type 2 diabetes, versus in only 19% of control subjects (Table 3).

#### Diameters of the pancreas-bound arteries

No difference was found between the 2 groups concerning the splenic, hepatic, gastroduodenal, inferior pancreatic, dorsal pancreatic, and greater pancreatic arteries diameters (data not shown).

The mean splenic artery diameter was higher in male than in female subjects (6.8 vs 5.6 mm, p < 0.0001). There was no correlation between splenic artery diameter and age, BMI, GFR, diabetes duration or HbA_1c_ (data not shown). However, in the patients with type 2 diabetes, the splenic artery diameter correlated with the pancreas volume (Spearman coefficient of correlation: 0.45, p = 0.0013). This correlation was not seen in the control subjects (Spearman coefficient of correlation: 0.09, p = 0.5). In the patients with type 2 diabetes but not in the control subjects, the splenic artery diameter also correlated with the hepatic artery diameter (Spearman coefficient of correlation 0.36 and 0.19, p = 0.01 and 0.2, respectively).

### Pancreas characterization

#### Pancreas volume and density

The median pancreas volume was not different between the patients with type 2 diabetes and the control subjects. Of note, the median pancreas volume was higher in male than in female subjects (62.5 cm^3^ vs 47 cm^3^, p = 0.005).

The median pancreas density was lower in the patients with type 2 diabetes than in the control subjects. As a comparison, the liver density was also lower in the patients with type 2 diabetes than in the control subjects (46.5 vs 53 HU, p = 0.0001) (Table 3).

### Multivariate analysis

We performed a multivariate logistic regression with adjustment for age, gender, BMI and the presence or absence of hypertension. After adjustment, the percentage of high calcium scores of the splenic artery and the abdominal aorta was still significantly higher in the patients with type 2 diabetes than in the control subjects, and the number of visible pancreas-bound branches and intrapancreatic arterial subdivisions was still significantly lower in the patients with type 2 diabetes than in the control subjects.

However, there was no statistically significant difference of pancreas density or liver density between the patients with type 2 diabetes and control subjects after adjustment.

### Pancreas characteristics according to renal function

As renal function is closely associated to vascular calcifications, we studied the relationships between renal function and the different characteristics of the pancreas. Thus, we compared the patients with or without renal failure, independently from their diabetes or control status. The subjects with renal failure were older than the subjects without renal failure (72.7 vs 63.8 years, p = 0.005). The subjects with renal failure tended to more often present with diabetes, but not significantly so. The splenic artery and abdominal aorta calcium scores, the number of pancreas-bound branches and intrapancreatic subdivisions, and the pancreas density did not differ significantly between subjects with or without renal failure (Additional file 1: Table S2). The pancreas volume tended to be lower in the subjects with renal failure than in the subjects without renal failure (43 cm^3^ vs 59 cm^3^, p = 0.05).

We also studied eGFR as a continuous variable. Age and BMI correlated negatively with eGFR (Spearman coefficient of correlation − 0.2 and − 0.27, p = 0.048 and 0.016, respectively).

The splenic artery and abdominal aorta calcium scores, the number of pancreas-bound branches and intrapancreatic arterial subdivisions, the pancreas volume and density did not correlate with eGFR (Additional file 1: Table S3 and Figure S1).

### Pancreas characteristics according to BMI

In control subjects, the pancreas volume was higher and the pancreas density was lower when the BMI was higher [Spearman coefficients of correlation: 0.6 (p = 0.0001) and − 0.37 (p = 0.03), respectively]. However, this relationship was not observed in patients with type 2 diabetes [Spearman coefficients of correlation: 0.21 (p = 0.16) and − 0.13 (p = 0.4), respectively].

As a comparison, the liver density was lower when the BMI was higher in control subjects and tended to be lower when the BMI was higher in patients with type 2 diabetes [Spearman coefficients of correlation: − 0.38 (p = 0.03) and − 0.29 (p = 0.05) respectively].

### Duration of diabetes

The 32 patients in whom the duration of diabetes was known were divided into two subgroups: 17 patients had a duration of diabetes of 0 to 10 years, while 15 patients had a duration of diabetes of more than 10 years. The patients with a long duration of diabetes were more often treated with insulin than the other patients. We did not observe a relationship between the volume or density of the pancreas and the duration of diabetes. Neither did we observe a relationship between the splenic artery calcium score, the abdominal aorta calcium score, the number of pancreatic branches or the number of intrapancreatic arterial subdivisions and the duration of diabetes (Table 4).

**Table 4:** Clinical characteristics of the patients according to diabetes duration

	0 to 10 years N = 17	> 10 years N = 15	p (univariate)	p (multivariate)
Diabetes duration, years: *mean* (*SD*)	5 (3.8)	20 (9.0)	< 0.0001	
Age, years: *mean* (*SD*)	63.2 (12.7)	62.3 (15.0)	0.85	
BMI, kg/m^2^: *mean* (*SD*)	27.7 (5.2)	28.8 (6.4)	0.6	
Insulin-treated patient, *n* (*%*)	6 (35)	12 (75)	0.04	0.049
Neuropathy, *n* (*%*)^a^	4 (33)	9 (60)	0.3	0.1
Nephropathy, *n* (*%*)^b^	4 (33)	5 (36)	1	0.5
Retinopathy, *n* (*%*)^c^	3 (27)	6 (46)	0.4	0.4
HbA_1c_, mmol/mol: *median* (*IQR*)^d^	56.0 (50–81)	70.0 (60–77)	0.3	0.6
HbA_1c_, *%: median* (*IQR*)^d^	7.3 (6.7–9.6)	8.6 (7.6–9.2)	0.3	0.6
Pancreas volume, cm^3^: *mean* (*SD*)	62.9 (39.6)	60.0 (27.7)	0.8	0.5
Pancreas density, *Hounsfield units: median* (*IQR*)^e^	29.6 (19.2–36.3)	27.7 (13.7–32.3)	0.9	0.6
Liver density, *Hounsfield units: median* (*IQR*)	50.0 (39.0–54.0)	48.0 (28.5–51.5)	0.3	0.2
Splenic artery calcium score *n* (*%*)				
0	13 (76)	7 (47)	0.2	0.2
1	2 (12)	4 (27)		
2 or 3	2 (12)	4 (27)		
Abdominal aorta calcium score, *n* (*%*)				
0	3 (18)	1 (7)	0.6	0.9
1	8 (47)	9 (60)		
2 or 3	6 (35)	5 (33)		
Number of pancreas-bound branches, *n* (*%*)				
0	6 (35)	5 (33)	1	0.9
1	4 (24)	4 (27)		
2 or 3	7 (41)	6 (40)		
Number of intrapancreatic arterial subdivisions, *n* (*%*)				
0	10 (59)	6 (40)	0.6	0.4
1	3 (18)	4 (27)		
2	4 (23)	5 (33)		

*SD* standard deviation. *IQR* interquartile range

^a^Available in 12 patients with a duration of diabetes of up to 10 years and in 14 patients with a duration of diabetes of > 10 years

^b^Available in 12 and 13 patients, respectively

^c^Available in 11 and 13 patients, respectively

^d^Available in 14 and 12 patients, respectively

^e^Pancreas density defined as the mean of 3 ROIs from the head, body and tail of the pancreas

When the duration of diabetes was analyzed as a continuous variable, insulin treatment was also the only variable that correlated with the duration of diabetes (Spearman coefficient of correlation: 0.47, p = 0.007): no correlation was found between duration of diabetes and vascular or pancreatic parameters.

## Discussion

To our knowledge, this is the first in vivo study to compare the specific vascularity and atherosclerosis of the pancreatic vessels between patients with type 2 diabetes and control subjects.

In the present study, patients with type 2 diabetes more often presented with calcifications of the splenic artery and with a less developed pancreatic arterial tree than control subjects matched for age and for gender. Pancreas volume was not different between the groups, and pancreas density was lower in patients with type 2 diabetes than in control subjects. Furthermore, the pancreas volume correlated with the splenic artery diameter. Correlation between pancreas volume and density and BMI was different in patients with type 2 diabetes and in control subjects.

The high frequency of splenic artery atherosclerosis in patients with type 2 diabetes may simply reflect a more atherogenic environment in these patients, hinted at by the higher frequency of hypertension, hypercholesterolemia and coronaropathy, and by the high frequency of atherosclerosis in the abdominal aorta. It could also reflect the level of macrovascular complications induced by diabetes.

However, we did not show a relationship between the duration of diabetes and the presence of splenic artery calcifications. Yet, this study was not powered to compare the clinical and pancreas characteristics according to diabetes duration, and this result could therefore be due to a lack of power of our study. It must also be noted that duration of diabetes was established according to patients and/or physician reports, and is known to be rather imprecise. Even in the UKPDS study [22], that studied newly-diagnosed patients with type 2 diabetes, more than 30% of patients already showed diabetic retinopathy. However, there is no other reliable way to know the real duration of diabetes in these patients, and even by using declarative duration of diabetes, studies have shown that global mortality, specific coronary heart disease mortality and microvascular complications are more frequent in patients with a long duration of diabetes than in patients with a recent diagnosis of diabetes [23–26]. Anyhow, even if pancreatic atherosclerosis was initially a mere vascular consequence of diabetes, one could imagine that it could later exert degrading effects on the endocrine pancreas due to pancreatic ischemia, and be part of a vicious circle that would worsen an already flailing endocrine function.

On another note, it could be argued that medial vascular calcification is a common feature in patients with type 2 diabetes [25, 27, 28], with active processes of vessels calcifications and remodeling [29, 30] and that we therefore cannot be sure that the calcifications found in the splenic artery are indeed atherosclerotic. However, different studies show that the presence of calcifications in the celiac trunk or in the superior mesenteric artery correlates with systemic calcified atherosclerosis, cardiovascular risk factors, all-cause mortality, and fatty liver. This could be in favor of an atherosclerotic process in these arteries [14, 31, 32].

We also showed that even after adjustment for age, gender, BMI and the presence of hypertension, the pancreatic arterial tree was less developed in patients with type 2 diabetes than in control subjects: the number of visible pancreas-bound branches and the number of intrapancreatic vessel divisions were lower in patients with type 2 diabetes than in control subjects. This is reminiscent of observations made about coronary epicardial vessels in patients with T2D [33] and concordant with the often-described aspect of “dead-tree” of arteries of lower limbs in diabetes, probably as a consequence of defective collateralization resulting from chronic hyperglycemia [34]. This also concurs with the many histopathological studies that show that lesions of the pancreatic vessels (from capillaries to little arteries) are more often seen in patients with type 2 diabetes than in control subjects [12, 13, 35–39]. This decreased vascularity in patients with type 2 diabetes could be associated with anomalies of pancreatic perfusion, in accordance with the study by Yu et al., that shows an increased endothelial permeability and a decreased plasma volume per unit of pancreas in patients with coronary disease and type 2 diabetes as compared to patients without type 2 diabetes [40]. Honka et al. also show that in obese patients, the pancreatic blood flow is inversely correlated with HbA_1c_ [41].

Besides, we compared the pancreas volume in patients with type 2 diabetes and in control subjects. The pancreas volume is known to be lower in patients with type 1 diabetes as compared to control subjects [42]. However, studies are discordant concerning the pancreas volume of patients with type 2 diabetes [43–48]. We did not find a difference in pancreas volume between the 2 groups. The pancreas volume of patients with type 2 diabetes in our study was very similar to the one described in previous works (61.5 cm^3^ vs 61.2 cm^3^ in a meta-analysis of 8 studies [46]), but the pancreas volume of the control subjects in our study was lower (60.7 vs 68 cm^3^ in subjects of the same mean age [46]). This could be explained by a relatively low BMI in the control subjects of our study (23.8 kg/m^2^ vs 27 kg/m^2^ in a meta-analysis [47]), as it is known that the pancreas volume increases with BMI [44, 49].

Of note, the pancreas volume tended to be lower in the patients who presented with renal failure. To our knowledge, this association has never been described. Supposedly, a common vascular factor could be at stake, and it would be interesting to investigate this correlation further in a future study.

We also confirmed that the pancreas density was lower in patients with type 2 diabetes than in control subjects, in accordance with existing data [45, 50, 51]. Because a lower density is associated with a higher fat content, the pancreas of the patients with type 2 diabetes was probably fattier than the controls’ [52]. Remarkably, pancreatic endocrine impairment seems to be associated with pancreatic fat both in patients with type 2 diabetes and without [53, 54], and pancreatic fat is associated with atherosclerosis and a higher cardiovascular risk in patients with type 2 diabetes [55, 56]. It must however be noted that there was no statistically significant difference of pancreas density between patients with type 2 diabetes and control subjects after adjustment for age, gender, BMI and presence of hypertension, and that this difference might thus be linked to confusion factors.

Similarly to what has been described in numerous studies, the pancreas volume and density [44, 49] correlated with BMI in control subjects. However, this relationship was lost for both parameters in patients with type 2 diabetes, which is discordant with the study by Saisho et al. [44]. In contrast, the association between liver steatosis and BMI was found both in patients with type 2 diabetes and in control subjects. We hypothesize that pancreatic atherosclerosis could have a specific impact on the pancreas of patients with type 2 diabetes. This idea is supported by the fact that, in the patients with type 2 diabetes but not in the control subjects, splenic artery diameter correlated positively with pancreas volume.

There are several weaknesses in this study. First, it is a retrospective study, and we only used the information available in the electronic records of the patients. Therefore, clinical data were often partially missing, leading to a lack of power for some of the analyses. For instance, BMI was not available for all of the patients and we were thus not able to match the patients and the control subjects for BMI. However, the calcium scores, number of pancreas-bound arteries and number of visible intrapancreatic arteries did not correlate with BMI in our study, either in the patients with type 2 diabetes or in the control subjects (data not shown) and the absence of matching is thus not likely to be a problem for the interpretation of results.

Moreover, by construction, a substantial part of the control subjects were oncology patients, since cancer follow-up is a frequent indication for performing abdominal CT. Consequently, the control subjects of our study had a lower BMI than what is usually seen in the literature, and might have presented with particularities of the abdominal CT as compared to other populations, e.g a lower liver density [57].

Besides, 1,5-anhydro-d-glucitol (1,5-ag) concentration, the level of which correlates with the severity of artery calcifications and constitute an independent risk factor of cardiovascular disease [58, 59], has not been measured in this study, which can be a limitation.

We used CT to assess pancreatic vessels rather than magnetic resonance imaging (MRI) because of the availability of CT and because of its spatial resolution. MRI could be of interest to study pancreatic perfusion using dedicated sequences of Dynamic Contrast Enhancement and software of perfusion quantification: for instance, Taso et al. showed that pancreatic basal blood flow was lower in patients with type 1 diabetes than in control subjects [60]. In addition, pancreatic MRI could be of value to assess fatty infiltration of the pancreas using dedicated q-dixon sequences in order to measure the fat fraction. Positron emission tomography (PET) has also been used to assess pancreatic perfusion, and it was shown that pancreatic perfusion was lower in patients with type 1 diabetes than in healthy individuals [61]. Nonetheless, MRI and PET are not routinely performed as first line imaging modality to explore pancreatic disorders.

For all these reasons, a prospective large-scale population study using other imaging modalities to assess pancreatic perfusion and fatty infiltration in patients with type 2 diabetes would be of use to confirm our results.

## Conclusions

Patients with type 2 diabetes presented with more calcifications of the splenic artery, and with a less dense pancreatic arterial tree than control subjects. Moreover, we showed that the relationship between BMI and pancreas characteristics seen in control subjects was not confirmed in patients with type 2 diabetes. We hypothesize that pancreatic atherosclerosis could have a specific impact on the pancreas of patients with T2D.

## Supplementary information

**Additional file 1: Table S1.** Causes for non-inclusion (absence of CT images with or without contrast for patients with T2D) or exclusion in patients with T2D and in control subjects. **Table S2.** Clinical and pancreas characteristics of the subjects with or without renal failure. **Table S3.** Glomerular filtration rate according to clinical and pancreas characteristics. **Table S4.** Diabetes duration according to clinical characteristics of the patients. **Figure S1.** Estimated glomerular filtration rate (eGFR) according to clinical and pancreas characteristics. **Figure S2.** Diabetes duration according to clinical and pancreas characteristics.

## Data Availability

The datasets used and/or analysed during the current study are available from the corresponding author on reasonable request.

## Abbreviation

BMI: Body mass index
