# Supplementary material for: Patients with type 2 diabetes present with multiple anomalies of the pancreatic arterial tree on abdominal computed tomography: comparison between patients with type 2 diabetes and a matched control group
Source: Cardiovasc Diabetol. 2020 Aug 5;19:122. doi: 10.1186/s12933-020-01098-1 (PMC7410152; doi:10.1186/s12933-020-01098-1)
Supplement: Supplementary file 1 — Additional file 1: Table S1. Causes for non-inclusion (absence of CT images with or without contrast for patients with T2D) or exclusion in patients with T2D and in control subjects. Table S2. Clinical and pancreas characteristics of the subjects with or without renal failure. Table S3. Glomerular filtration rate according to clinical and pancreas characteristics. Table S4. Diabetes duration according to clinical characteristics of the patients. Figure S1. Estimated glomerular filtration rate (eGFR) according to clinical and pancreas characteristics. Figure S2. Diabetes duration according to clinical and pancreas characteristics. [file 12933_2020_1098_MOESM1_ESM.docx]

|  | **Patients with T2D** | **Control subjects** |
| --- | --- | --- |
| Type 1 diabetes | 4 | 1 |
| Other types of diabetes (MODY, cystic fibrosis) | 3 | 2 |
| Acute pancreatitis in the 6 months prior to the scan | 2 | 3 |
| Chronic pancreatitis | 4 | 2 |
| Pancreatic adenocarcinoma/ history of pancreatic surgery (without precisions) | 11 | 10 |
| Other pancreatic anomalies (intraductal papillary mucinous tumors of the pancreas, pancreatic metastases of other cancers…) | 3 | 20 |
| Liver or biliary disease (Hepatocellular carcinoma, advanced cirrhosis, cholangiocarcinoma, biliary prosthesis) | 8 | 79 |
| Cardiorespiratory arrest or multi-visceral failure in the month prior or in the week following the CT scan | 4 | 4 |
| Absence of recent medical record | 2 | 39 |
| CT scan: performed without injection of contrast media or performed only after injection of contrast media | 9 | NA |
| Type 2 Diabetes | NA | 17 |

**Table S1. Causes for non-inclusion (absence of CT images with or without contrast for patients with T2D) or exclusion in patients with T2D and in control subjects.**

|  | Subjects with renal failure (12) | Subjects without renal failure (82) | p |
| --- | --- | --- | --- |
| **General characteristics** |  |  |  |
| Male, *n (%)* | 7 (58) | 53 (65) | 0.7 |
| Age, *years: mean (SD)* | 72.7 (13.2) | 63.8 (12.3) | 0.005 |
| BMI, *kg/m^2^: mean (SD)*^a^ | 27.9 (6.1) | 25.7 (5.8) | 0.2 |
| Glomerular filtration rate : *mL/min: mean (SD)*  Patients with T2D, *n (%)* | 47.2 (13.5)  9 (75) | 94.8 (25.4)  39 (48) | <0.0001  0.08 |
| **Diabetes characteristics (only in patients with T2D)** |  |  |  |
| HbA_1c,_ *mmol/mol: median (IQR)*^b^ | 60 (45-86) | 58 (51-76) | 0.4 |
| HbA_1c,_ *%:* *median (IQR)*^b^ | 7.6 (6.3-10.5) | 7.5 (6.8-9.1) | 0.4 |
| Diabetes duration, *years: median (IQR)*^c^ | 9 (3-11) | 12 (6-20.5) | 0.3 |
| Insulin use, *n (%)* | 5 (56) | 14 (36) | 0.5 |
| **Pancreas and vessels characteristics** |  |  |  |
| Pancreas volume, *cm^3^: mean (SD)* | 45.8 (22.7) | 62.9 (29.6) | 0.05 |
| Pancreas density, *Hounsfield units : median (IQR)* | 21.8 (8.7-35.1) | 30.5 (23.9-38) | 0.1 |
| Liver density, *Hounsfield units : median (IQR)* | 48.5 (41-53.5) | 49.5 (42-53.3) | 0.7 |
| Splenic artery calcium score, *n (%)*  0  1  2 or 3 | 6 (50)  3 (25)  3 (25) | 62 (76)  14 (17)  6 (7) | 0.09 |
| Abdominal aorta calcium score, *n (%)*  0  1  2 or 3 | 0 (0)  6 (50)  6 (50) | 17 (21)  34 (42)  31 (38) | 0.2 |
| Number of pancreas- bound arteries, *n (%)*  0  1  2 or 3 | 6 (50)  2 (17)  4 (33) | 19 (32)  15 (18)  48 (59) | 0.1 |
| Number of intrapancreatic arterial subdivisions, *n (%)*  0  1  2 | 6 (50)  3 (25)  3 (25) | 29 (35)  21 (26)  32 (39) | 0.6 |

**Table S2. Clinical and pancreas characteristics of the subjects with or without renal failure.**

Renal failure (RF) defined as a eGFR <60mL/min/1.73m^2^. T2D : Type 2 Diabetes. BMI : Body Mass Index. SD : Standard Deviation. IQR : Interquartile Range.

^a^Available in 12 subjects with RF and 64 subjects without RF. ^b^Last HbA_1c_ in the medical file. Only the HbA_1c_ from the patients with T2D were used so as to compare homogenous groups. Available in 8 patients with RF and 26 patients withtout RF. ^c^Available in 7 patients with RF and 25 patients without RF.

|  | Estimated glomerular filtration rate (mean, SD) | p |
| --- | --- | --- |
| **Gender**  Male  Female  **Type 2 diabetes**^a^  Yes  No | 85.2 (24.4)  93.1 (28.9)  85.9 (30.3)  94.9 (23.6) | 0.2  0.1 |
| **Insulin use**^b^  Yes  No | 81.7 (34.7)  88.6 (27.3) | 0.4 |
| **Pancreas and vessels characteristics** |  |  |
| Splenic artery calcium score^c^  0  1  2 or 3 | 90.0 (23.1)  97.3 (35.2)  78.9 (39.4) | 0.3 |
| Abdominal aorta calcium score^d^  0  1  2 or 3 | 99.2 (17.1)  86.3 (29.0)  90.5 (29.2) | 0.3 |
| Number of pancreas- bound arteries^e^  0  1  2 or 3 | 81.5 (27.4)  89.5 (19.2)  94.7 (29.1) | 0.1 |
| Number of intrapancreatic arterial subdivisions^f^  0  1  2 | 85.6 (25)  91.5 (32.2)  94.1 (26.4) | 0.4 |

**Table S3. Glomerular filtration rate according to clinical and pancreas characteristics**

^a^Available in 48 patients with T2D and 46 control subjects. ^b^ Availabe in 29 patients taking insulin and 19 patients not taking insulin. ^c^Available in 68, 17 and 9 patients, respectively. ^d^Available in 17, 40 and 37 patients, respectively. ^e^Available in 25, 17 and 52 patients, respectively. ^f^Available in 35, 24 and 35 patients, respectively.

** Figure S1. Estimated glomerular filtration rate (eGFR) according to clinical and pancreas characteristics.**

eGFR (MDRD formula) in mL/min/1.73m^2^. For all figures, a Spearman coefficient of correlation (r) and p-value (p) were calculated. BMI : body mass index.

a.eGFR according to age in years. b.eGFR according to body mass index in kg/m^2^. c.eGFR according to HbA_1c_ (in %) in patients with type 2 diabetes. d.eGFR according to diabetes duratin (in years) in patients with type 2 diabetes. e.eGFR according to pancreas volume in cm^3^. f.eGFR according to mean pancreas density as measured on images without contrast, in Hounsfield Units. g.eGFR according to liver density as measured on images without contrast, in Housfield Units.

|  | Diabetes duration (median, IQR) | p |
| --- | --- | --- |
| Insulin use^a^  Yes  No | 12 (9.5-20.5)  7 (0.5-12) | 0.0085 |
| Neuropathy^b^  Yes  No | 12 (8-24)  9 (0.25-16) | 0.098 |
| Nephropathy^c^  Yes  No | 11 (5.5-15)  10.5 (2.75-21.25) | 0.9 |
| Retinopathy^d^  Yes  No | 13 (8.5-22)  9 (2-15) | 0.2 |
| Splenic artery calcium score^e^  0  1  2 or 3 | 9 (4.25-12.75)  13 (5.25-23.75)  15 (5.25-28.75) | 0.5 |
| Abdominal aorta calcium score^f^  0  1  2 or 3 | 9 (8.25-11.25)  12 (3-18.5)  10 (7-26) | 0.8 |
| Number of pancreas-bound branches^g^  0  1  2 or 3 | 10 (3-19)  10 (7.5-24.75)  10 (2.75-16.5) | 0.8 |
| Number of intrapancreatic arterial subdivisions^h^  0  1  2 | 8.5 (2.25-17.75)  13 (9-7)  11 (7-13.5) | 0.3 |

**Table S4. Diabetes duration according to clinical characteristics of the patients**

IQR : Interquartile Range

^a^Available in 15 and 17 patients, respectively. ^b^Available in 13 and 13 patients, respectively. ^c^Available in 9 and 16 patients, respectively. ^d^Available in 9 and 15 patients, respectively. ^e^Available in 20, 6 and 6 patients, respectively. ^f^Available in 4, 17 and 11 patients, respectively. ^g^Available in 11, 8 and 13 patients, respectively. ^h^Available in 16, 7 and 9 patients, respectively.

**Figure S2. Diabetes duration according to clinical and pancreas characteristics.**

r = Spearman coefficient of correlation, p = p value of correlation.
